# Our New York Letter

**Published:** 1889-11

**Authors:** M. B. Hutchins

**Affiliations:** New York


### Correspondence.

### OUR NEW YORK LETTER.

NEW YORK, October, 1889.

The brief sensation over the Brown-Sequard "Elixir" seems to have subsided, some substantial gentlemen of the profession having, however, gone so far as to make experiments with the fluid; and at least two well known M. Ds. had a rather sharp newpaper controversy on the subject. Practical men early dismissed the subject, saying the famous Brown-Sequard was in his dotage.

It is to be regretted that some one of New York's great dermatologists did not attend the International Congress of Dermatology and Syphilis in Paris. Distinguished men from other countries were present, but the United States were only partially represented. Many distinguished questions were discussed and one who reads the report of proceedings can at least approximate a definite conclusion as to the classification of certain diseases whose designation is not yet settled. The few who stand alone and unchanged in their views must finally yield to the force of the majority, or else lose caste as liberal and substantial exponents of dermatology. Dermatologists seem to have more disagreements than any other class of specialists. The other day a prominent Brooklyn dermatologist remarked that he yet had eleven years of his allotted time to live. A friend remarked, "then you had better resign from the New York Dermatological Society," thinking, perhaps, that eleven years was longer than a man could stand the "fights" of that body.

At the meeting of the Academy of Medicine of October 3d, Dr. Chas. L. Dana read a paper upon the pathological anatomy of chorea, reported a case and the autopsy. Mentioned, in passing, the occurrence of fibrinous deposits upon the valves of the heart, (chiefly mitral), which were formed in ninety per cent. of the cases examined. The disease developed at the age of six in his case. Attacks were epileptoid in character and "choreic movements" were such as to keep the patient in bed. History of trephining with the hope of relieving symptoms. Dr. Dana thought so-called epileptoid attacks "belonged rather to hysterical type." Condition improved under administration of chloral hydrate and stimulants. Chloral afterwards suspended and physostigma given. Patient died of pneumonia. Skull found thick; no excess of meningeal fluid; brain appeared normal, save presence of slight congestion and superficial softening. Portions of brain showed honey-combed appearance, spaces of which were either empty or filled with blood vessels. No genuine arteritis found but there was present perivascular infiltration. "Temporal" lobes showed severer grades of meningitis, honey-combed and an increase of neuroglia cells. Symptoms of arteritis present in optic thalamus, and other portions of small extent. Degeneration similar to that recently described by European writers. In the discussion, Dr. M. A. Starr went quite fully into the subject and, though the specific organism has not yet been definitely agreed upon, thinks the disease will yet prove of parasitic origin. Dr. Mary Putnam Jacobi doubted the germ theory, saying it was strange the "organism" said to produce the disease should lie dormant until choreic symptoms arose, following a fright or a fall down stairs." She quoted recent literature upon the subject, at some length. One of the speakers, as well as Dr. Dana himself, inclined to believe in the "humoral" theory of the disease. The President, Dr. Loomis, thought the condition would be proven of parasitic origin, and remarked that the brain conditions described by Dr. Dana were found in post mortems of cases dying of other diseases. Dr. Dana denied this and regretted that Dr. Loomis had failed to mention the diseases. Dr. Dana's paper is another step towards a better understanding of the nature of chorea, but it is clear that we have not yet learned the true cause.

Two interesting persons present were Dr. Mary Putnam Jacobi, and Dr. O'Dwyer of intubation fame. The former is a lady of apparently middle age, medium size. Her features are plain; nose rather broad and "flat." She dresses plainly and has the appearance of a staid matron, devoted only to her household affairs, instead of being perhaps the leading lady physician in this country, and one whose fame is greater than that of her distinguished husband, Dr. A. Jacobi. Dr. O'Dwyer, whose name is so often heard in connection with laryngeal intubation, is tall, rather slender, dresses plainly, having much the appearance of a Georgia village lawyer or preacher. His face and prominent Roman nose indicate stability and firmness, while the picture is completed by a high forehead, topped with grayish hair, thick and rather pompadour in style of cut; and finally a full beard, cut close on the cheeks. The doctor appears, as he is said to be, modest and retiring. Dr. Dillon Brown, who did much to aid Dr. O'Dwyer in bringing out intubation, is youthful looking, energetic and is rapidly rising in the profession.

Dr. J. A. Fordyce describes, in the last number of the "*Journal of Cutaneous and Genito-Urinary Diseases*," a case in which purpura and psoriasis were combined. A patient has just left the Skin and Cancer Hospital who presented the rare combination of an eczematous and purpuric state. Purpura and psoriasis might occur from their presumptive cause, the rheumatic diathesis. Eczema has been described as occurring in nearly every disordered systemic state.

A patient, a young man aged 17, entered the hospital with a disease of the following description: Affected portions on the face, posterior part of scalp, neck, chest, back, arms, with a few isolated lesions on body and legs. Besides numerous "blackheads" (comedones), the face showed characteristics of the worst form of *acne vulgaris*, with numerous sebaceous abscesses, and over these severe lesions a diffuse, thin crusting—as of dried sulphur ointment. Upon neck, scalp, arms, chest and shoulders usual acute lesions, comedones and some crusting. Numerous vesicles and bullae form constantly in these situations, some springing up apparently without a sign of inflammatory action, but the majority showing pale red areola. Contents apparently sero-pus from the beginning of even the pin head sized lesions. A few showed central comedo. Patient markedly cachetic. Dr. G. H. Fox was inclined to the diagnosis of Duhring's dermatitis herpetiformis; Dr. G. T. Elliott diagnosed pustular eczema. Dr. P. A. Morrow considered disease either a bromide or an iodide eruption—preferably the latter. At the time there was no history of the ingestion of either drug, though it has since been learned that the boy was taking iodide of potassium—gr. xv per day. Drs. L. D. Bulkley, Robinson, Bronson and Sherwell (of Brooklyn), said the disease was acute cachecticorum. Of course those who made other diagnoses excluded the acne condition of face. The presence of comedones in such an unusual situation as the forearm and cachexia of patient adds weight to the evidence in favor "acute cachecticorum."

A woman in the hospital with undoubted tubercular syphilis and a clear "specific" history could speak only in a whisper. Dr. Geo. M. Lefferts, consulting laryngologist, made an examination. Epiglottis and glottis the seat of a number of tubercular or nodular lesions, vocal cords affected and partially destroyed. Condition present three months. Appearance and duration without ulceration led the doctor to diagnose lupus of the larynx. Dr. Lefferts' reputation considered on the one side, and the undoubted syphilitic character of the general disease on the other, led to question as to the probability of two distinct diseases being present in this case.

A young woman had "initial lesion" of the lip. When the lip had nearly healed under local treatment alone (and after three weeks' duration), there was still no primary eruption. (No internal treatment was given.) Eight weeks after occurrence of chancre, the maculo-papular eruption occurred. Treatment then begun and, according to the woman's statement, *rather pushed*, as she said she took forty-five grains of calomel by mistake. At any rate the eruption faded rapidly.

A patient with papulo-pustular (small) syphilitic eruption was treated for two or three weeks with inunctions of ungt. hydrarg. ʒi. morning and night. (There was also excessive enlargement and induration of lymphatic glands over right side of inferior maxilla.) Inunctions had scarcely any effect, patient even having during the time symptoms attributable to "secondary fever." Mixed treatment being given, all symptoms began rapid improvement. Another case with chronic syphilitic glossitis received no benefit from several months' administration of "mixed," but the tongue got well in a few weeks after regular use of the inunction method.

A German woman had a peculiar scar over upper border of sternum, it being smooth as if burned, but having a small opening in the center through which was apparently raw adipose tissue. Surrounding this scar, especially involving insertion of sterno-mastoid muscle, was a dense infiltration. Opening probed, giving feeling of necrosis when sternum was touched. A few syphilitic scars on chest. "No history." An incision was made and preparations to scrape away necrotic bone. "Gummy" infiltrate "scraped" out and then the supposed necrosis was found to be a simple roughening of the bone. The surgeon aptly remarked: "Much ado about nothing," put two stitches in the lower end of incision, dusted in the iodoform, packed with gauze, and left the wound to granulate.

An Italian, aged 60, was seen by a young M. D. of the city medical service, and the condition of skin present suggested leprosy to the doctor's mind. The legs and feet especially involved. Irregular patches and tubercles of infiltration, brown or purple in color, some even tending to blackness; no ulceration; pain in feet intense. Diagnosis, "Multiple sarcoma."

Funk, in "*Monatschrifte für Praktische Dermatologie*," reports a number of these cases cured by hypodermic injections of arsenical solution. A case, not very severe, which I have seen, was thus treated. I have heard that the patient died of abscess.

There is an old Irish woman who comes here to the clinic who has at least a half dozen epitheliomata on scalp, face and upper part of body. Some are nearly healed, and none very active; patient's health apparently unaffected.

M. B. HUTCHINS, M. D.

