# A Correction

**Published:** 1889-12

**Authors:** 


A CORRECTION.—In New York letter of October, page 570 lines 11 and 15, "*acne cachicticorum*" is printed "*acute cachictico-rum.*"

